# Active Transport Can Greatly Enhance Cdc20:Mad2 Formation

**DOI:** 10.3390/ijms151019074

**Published:** 2014-10-21

**Authors:** Bashar Ibrahim, Richard Henze

**Affiliations:** 1 1 Al-Qunfudah University College, Umm Al-Qura University, 1109 Makkah Al-Mukarramah, Saudi Arabia; 2 2 Al-Qunfudah Center for Scientific Research (QCSR), 21912 Al-Qunfudah, Saudi Arabia; 3 3 Bio Systems Analysis Group, Institute of Computer Science, Jena Center for Bioinformatics and Friedrich Schiller University, 07743 Jena, Germany; E-Mail: richard.henze@uni-jena.de

**Keywords:** spindle assembly checkpoint, chromosome segregation, systems biology of mitosis, simulation, modeling

## Abstract

To guarantee genomic integrity and viability, the cell must ensure proper distribution of the replicated chromosomes among the two daughter cells in mitosis. The mitotic spindle assembly checkpoint (SAC) is a central regulatory mechanism to achieve this goal. A dysfunction of this checkpoint may lead to aneuploidy and likely contributes to the development of cancer. Kinetochores of unattached or misaligned chromosomes are thought to generate a diffusible “wait-anaphase” signal, which is the basis for downstream events to inhibit the anaphase promoting complex/cyclosome (APC/C). The rate of Cdc20:C-Mad2 complex formation at the kinetochore is a key regulatory factor in the context of APC/C inhibition. Computer simulations of a quantitative SAC model show that the formation of Cdc20:C-Mad2 is too slow for checkpoint maintenance when cytosolic O-Mad2 has to encounter kinetochores by diffusion alone. Here, we show that an active transport of O-Mad2 towards the spindle mid-zone increases the efficiency of Mad2-activation. Our *in-silico* data indicate that this mechanism can greatly enhance the formation of Cdc20:Mad2 and furthermore gives an explanation on how the “wait-anaphase” signal can dissolve abruptly within a short time. Our results help to understand parts of the SAC mechanism that remain unclear.

## 1. Introduction

Correct distribution of the replicated genome during mitosis is essential in all proliferating cells. In order to accomplish this, the cell must guarantee that each chromosome has established a tight bipolar attachment to the spindle apparatus before sister-chromatid separation is initiated in anaphase. The mitotic Spindle Assembly Checkpoint (SAC; [1]) is a surveillance mechanism that delays the onset of anaphase until all chromosomes have made their attachments to the mitotic spindle. Even one misaligned chromosome is sufficient to keep the checkpoint active, yet the mechanism by which this is achieved is still elusive. It is thought that unattached or misaligned kinetochores catalyze the formation of a “wait-anaphase” signal. This signal eventually diffuses to counter the activation of the ubiquitin ligase anaphase promoting complex/cyclosome (APC/C) by its co-activator Cdc20 (Cell Division Cycle 20 homolog [2]). The activation of APC by Cdc20 triggers chromosome segregation by ubiquitination of securin and cyclin B [3,4,5,6] (for review see [7]). A dysfunction of the SAC may lead to aneuploidy [8,9] and furthermore its reliable function is important for tumor suppression [10,11,12].

The core proteins involved in the SAC, conserved in all eukaryotes, are MAD (“Mitotic Arrest Deficient”; Mad1, Mad2, and Mad3 (in humans BubR1) [13]) and BUB (“Budding Uninhibited by Benzimidazole”; Bub1, and Bub3 [14]). These proteins work to regulate APC activity and its co-activator Cdc20. In addition to these two core proteins, the SAC also involves several other components that participate in essential aspects of this mechanism. Among these components are Aurora-B [15] and the “Multipolar spindle-1” protein (Mps1) [16]. These two proteins are required for SAC signal amplification and the formation of the Mitotic Checkpoint Complex (MCC). Moreover, several other components, involved in carrying out essential aspects of the SAC mechanism, have been identified in higher eukaryotes. Those are for example the RZZ complex [17,18] which is composed of “Rough Deal” (Rod) [19,20], Zeste White 10 [20,21,22,23] and Zwint-1 [24].

The Cdc20-binding protein Mad2 was suggested as a candidate for the “wait-anaphase” signal, as it is stabilized in a conformation with increased affinity to Cdc20 specifically at unattached kinetochores. The resulting Cdc20:C-Mad2 complex is diffusible and can bind to a complex of Bub3 and BubR1 to form the possibly transient mitotic checkpoint complex (MCC), which is a potent inhibitor of the APC. Additional, Cdc20:C-Mad2 can bind directly to the APC and form an inactive complex [25].

Several mathematical models have been developed during the last decade to evaluate possible mechanisms for signal generation and propagation. Doncic *et al*. [26] as well as Sear and Howard [27] analyzed simple spatial models of potential checkpoint mechanisms with focus on budding yeast or metazoan, respectively. They observed theoretically that a diffusible signal can generally account for checkpoint operation. A more detailed model of the human SAC, including many of the confirmed interactions, has been proposed by Ibrahim *et al*. [28,29,30]. It is based on the “Mad2 template” model by De Antoni *et al*. [31] and has been supported with *in-vitro* experiments by Simonetta *et al*. [32]. Lohel *et al*. [33] achieved an improvement of this model by taking into account species localization and binding sites at kinetochores.

Here, we discuss a spatial model of the SAC, assuming the rate of Cdc20:C-Mad2 complex formation as the key regulatory factor for SAC activation and maintenance. Therefore, we develop a 3-dimensional model of one mitotic cell and its last unattached kinetochore, based on the “Mad2 template” model [31]. All core proteins and their reactions are enabled with *in-vitro* measured concentrations and interaction rates. Thus, our simulations give a reliable insight into the behavior of the real SAC system. To mimic the majority of experimental findings we mainly focus on human data but also use budding yeast results for a comparison. Taking into account the fact that animals undergo an “open” mitosis, where the nuclear envelope breaks down before the chromosomes separate, while Saccharomyces cerevisiae (yeast) undergoes a “closed” mitosis, where chromosomes divide within an intact cell nucleus. To overcome the issue of a “closed” mitosis for the budding yeast Saccharomyces cerevisiae model, we define the nucleus as a compartment model which is exactly the meaning of a “closed” mitosis. However, from a theoretical point of view if the diffusion is sufficient for the whole cell this implies that the diffusion must be sufficient for a smaller sub-volume too, like the nucleus.

First, we implemented the full “Mad2 template” model, including amplification, to check whether this has an effect on the formation of Cdc20:C-Mad2. As the diffusion rate of O-Mad2 is not certainly known yet, we estimated a suitable value. Therefore, we assumed that a high concentration of Cdc20:C-Mad2 supports the SAC, varied the diffusion of free Mad2 in a range from 0.0–50.0 *µ*m^2^s^−^^1^ and used the amount of formed Cdc20:C-Mad2 as score. Furthermore, the idea of an active O-Mad2 transport came up to support the formation of Cfdc20:C-Mad2 at the kinetochore [27]. We show that the effective Cdc20:C-Mad2 formation rate does not only depend on its chemical kinetics, but in addition requires a high flux of free Mad2 towards the spindle mid-zone in human cells.

## 2. The Model

### 2.1. Mad2 Template Model

DeAntoni *et al*. suggested a simple SAC model [31], which describes the mechanism of Mad2 recruitment to the kinetochore and the transport of Mad2 to Cdc20. This model got known as the “Mad2 template” model as Mad1 and Mad2 form a compound in the vicinity of a kinetochore to activate Cdc20. The dynamic of the six species O-Mad2, Mad1:C-Mad2, Mad1:C-Mad2:O-Mad2^*^, Cdc20, Cdc20:C-Mad2, and Cdc20:C-Mad2:Mad2* is described by the reaction Equations (1)–(5). The Equations (1)–(3) represent the basic “template model”, while the additional Equations (4) and (5) refer to the effect of amplification (autocatalytic loop).

(1) $$\text{Cdc20}+\text{O-Mad2}\overset{{\text{k}}_{1}}{\underset{{\text{k}}_{-1}}{⇌}}\text{Cdc20:C-Mad2}$$

(2) $$\text{Mad1:C-Mad2}+\text{O-Mad2}\overset{{\text{k}}_{2}}{\underset{{\text{k}}_{-2}}{⇌}}{\text{Mad1:C-Mad2:Mad2}}^{\text{*}}$$

(3) $${\text{Mad1:C-Mad2:Mad2}}^{\text{*}}+\text{Cdc20}\overset{{\text{k}}_{3}}{\underset{{\text{k}}_{-3}}{⇌}}\text{Mad1:C-Mad2}+\text{Cdc20:C-Mad2}$$

(4) $$\text{Cdc20:C-Mad2}+\text{O-Mad2}\overset{{\text{k}}_{4}}{\underset{{\text{k}}_{-4}}{⇌}}{\text{Cdc20:C-Mad2:Mad2}}^{\text{*}}$$

(5) $$\text{Cdc20:C-Mad2}+\text{O-Mad2}\overset{{\text{k}}_{5}}{\underset{{\text{k}}_{-5}}{⇌}}{\text{Cdc20:C-Mad2:Mad2}}^{\text{*}}$$

The biochemical reactions of SAC activation and maintenance mechanism can be divided into a kinetochore and a cytosolic part. The former serves to communicate the attachment status of each kinetochore to the remainder of the cell while the latter one accounts for the actual inhibition of the APC.

#### 2.1.1. Mad2-Activation and Its Function in Sequestering Cdc20

Mad2-activation at the kinetochore is commonly seen as the central part of the SAC mechanism. According to the “Mad2 template” model, Mad2 in its open conformation (O-Mad2) is recruited to unattached kinetochores by Mad1-bound Mad2 in its close conformation (C-Mad2) to form the ternary complex Mad1:C-Mad2:O-Mad2^*^ (*cf.* Reaction (2)). In this complex O-Mad2^*^ is the “activated” Mad2, *i.e.*, it is stabilized in a conformation which can interact with Cdc20 to form Cdc20:C-Mad2 (*cf.* Reaction (3)). The kinetic rate coefficients for this interactions have been determined *in-vitro* by [32] (*cf.* Table 1). In addition, O-Mad2 can likewise be activated by Cdc20 to increase Cdc20:C-Mad2 autocatalytically [32] (*cf.* Reaction (1)).

#### 2.1.2. Autocatalytic Amplification of Cdc20:C-Mad2 Formation

The addition of reactions (4) and (5) results in reaction (1). For that reason the amplification is also known as autocatalytic loop. The contribution of this pathway is minor with the kinetic data given in [32] (*cf.* Table 1). The presence of a highly contributing autocatalytic loop would also counteract the checkpoint deactivation and is therefore not desirable for living cells from a theoretical point of view [28]. While Mad1:C-Mad2 only exists at the kinetochore, Cdc20:C-Mad2 can be seen as structural equivalent of it in the cytosol. Thus, Cdc20:C-Mad2 converts more O-Mad2 into Cdc20 bound C-Mad2 (*cf.* Reaction (4)) and subsequently binds Cdc20 (*cf.* Reaction (5)). This implies that the amplification has just a minor contribution to the formation of Cdc20:C-Mad2, as also observed by [28].

**Table 1:** Parameters for the mitotic spindle assembly model.

	Parameter	Human	Budding Yeast	Remarks
**Rate constants:**	k_1_	1.00 *×* 10^−^^3^ *µ*M*^−^*^1^s*^−^*^1^	4.83 *×* 10^−^^5^ *µ*M*^−^*^1^s*^−^*^1^	[34]/[32]
	k_2_	2.00 *×* 10^−^^1^ *µ*M^−^^1^s^−^^1^	3.00 *×* 10^−^^1^ *µ*M^−^^1^s^−^^1^	[35]/[32]
	k_3_	1.00 *×* 10^2^ *µ*M*^−^*^1^s*^−^*^1^	3.00 *×* 10^−^^3^ *µ*M*^−^*^1^s*^−^*^1^	[28]/[32]
	k_4_	1.00 *×* 10^−^^2^ *µ*M*^−^*^1^s*^−^*^1^	*NA*	[28]
	k_5_	1.00 *×* 10^2^ *µ*M*^−^*^1^ s*^−^*^1^	*NA*	[28]
	k_−__1_	1.00 *×* 10^−^^2 ^s*^−^*^1^	4.83 *×* 10^−^^6^ s*^−^*^1^	[28]/[32]
	k_−__2_	2.00 *×* 10^−1^s^−^^1^	4.50 *×* 10^−^^1^ s^−^^1^	[35]/[32]
	k_−__3_	0.00 s^−^^1^	2.00 *×* 10^−^^1 ^*µ*M^−^^1^s^−^^1^	[28]/[32]
	k_−__4_	3.00 *×* 10^−^^2^ s^−^^1^	*NA*	[28]
	k_−__5_	0.00 s^−^^1^	*NA*	[28]
**Initial amount:**				
	Cdc20	0.22 *µ*M	0.1 *µ*M	[36,37]/[32]
	O-Mad2	0.15 *µ*M	0.2 *µ*M	[35]/[32]
	Cdc20:C-Mad2	0 *µ*M	0 *µ*M	[29]/[33]
	Mad1:C-Mad2	0.05 *µ*M	0.00616 *µ*M	[31]/[32]
	Mad1:C-Mad2:Mad2^*^	0 *µ*M	0 *µ*M	[31]/[32]
****	Cdc20:C-Mad2:Mad2^*^	0 *µ*M	*NA*	[31]
**Diffusion constants:**				
	Cdc20	19.5 *µ*m^2^s^−^^1^	19.5 *µ*m^2^s^−^^1^	[38]
	O-Mad2	0.0 *−* 50.0 *µ*m^2^s^−^^1^	0.0 *−* 50.0 *µ*m^2^s^−^^1^	
	Cdc20:C-Mad2	0.0 *−* 14.0 *µ*m^2^s^−^^1^	0.0 *−* 14.0 *µ*m^2^s^−^^1^	
	Mad1:C-Mad2	0	0	
	Mad1:C-Mad2:Mad2^*^	0	0	
	Cdc20:C-Mad2:Mad2^*^	0.0 *−* 11.0 *µ*m^2^s^−^^1^	*NA*	
**Environment:**				
	radius of the kinetochore	0.1 *µ*m	0.015 *µ*m	[39]/[40,41]
	radius of the cell	10 *µ*m	2 *µ*m	[42]/[43]

#### 2.1.3. APC Inhibition

Although Cdc20:C-Mad2 can bind to and inhibit APC directly, its inhibitory potency increases greatly in synergy with the Bub3:BubR1 complex [34]. It was shown that Cdc20:C-Mad2 together with Bub3:BubR1 forms the tetrameric mitotic checkpoint complex MCC which is a powerful inhibitor of APC [44]. Also the ternary complex Bub3:BubR1:Cdc20 alone is an effective inhibitor of APC [45]. However, the rate of its uncatalyzed formation in the cytosol is slow [44]. The formation of Bub3:BubR1:Cdc20 is accelerated in the presence of unattached chromosomes [46] and it might be that MCC forms as an intermediate complex from which O-Mad2 rapidly dissociates [45,46,47]. The demanding mechanisms for binding the inhibitory complexes to the APC are subject to current research. Despite its possibly transient nature in Bub3:BubR1:Cdc20 formation, MCC has been found stably bound to APC in mitosis [48].  The MCC might form more stably at unattached kinetochores and recruit APC from the cytoplasm. The MCC sub-complexes Bub3:BubR1:Cdc20 and Cdc20:C-Mad2 can bind to the APC independently from unattached kinetochores although their binding might be facilitated in a kinetochore-dependent manner [45,46,47]. Also, the MCC might bind to APC in a kinetochore-independent manner to eventually inhibit APC by releasing O-Mad2, forming the stable Bub3:BubR1:Cdc20:APC complex. The mitotic checkpoint factor 2 protein (MCF2) is a highly potent APC:inhibitor, yet the mechanism of binding to the APC and its regulation is still unknown [49,50]. With the exception of MCF2, all complexes inhibiting APC rely on the presence of Cdc20:C-Mad2, which requires unattached kinetochores for adequately fast formation. We can categorize the Cdc20:C-Mad2 complex as “interface” which links signaling from unattached kinetochores to APC inhibition. Hence, in this article we focus our analysis on the dynamics of the Cdc20 and Mad2 concentrations. We did not consider APC explicitly due to the fact that there are other direct inhibitory pathways for APC, like the MCC or MCF2 complexes. Including solely an APC reaction to Cdc20 would provide no additional information from the simulation. Additionally, there is less kinetic data available on such level of detail. However, we think future work of detailed spatial APC models is required.

### 2.2. Mathematical Treatment and Simulation

#### 2.2.1. Reaction-Diffusion-Convection System

The models reactions are translated to mathematical language of coupled ordinary differential equations (ODEs). Adding a second spatial-derivative as diffusion term transforms the system in coupled partial differential equations (PDEs) known as *Reaction-Diffusion system*:

(6) $$\frac{\partial [Ci]\;}{\partial t}=\underset{\text{Diffusion}}{\underset{︸}{D_{i}\nabla^{2}[C_{i}]}}+\underset{\text{Reaction}}{\underset{︸}{R_{j}(\{[C_{i}]\},\text{P})}}.$$

where [*Ci*] refers to the concentration of species *i* = {1,...,6}. The first term on the right hand side represents the diffusion and the second one represents the biochemical reactions *Rj* = {*R*_1_, ..., *R*_5_} where species *i* is involved. The constant D*i* denotes its species diffusion. The operator ∇ refers to the spatial gradient ($\vec{\nabla }=\vec{e_{x}}\frac{\partial }{\partial x}+\vec{e_{y}}\frac{\partial }{\partial y}+\vec{e_{z}}\frac{\partial }{\partial z}$), *t* adverts to the time and P symbolizes phenomenological *∂y* parameters. The full reaction-diffusion system, including six species and five reactions, can be written with PDEs as following:

(7) $$\frac{\partial \text{[Cdc20]}}{\partial t}{\text{=D}}_{\text{1}}\nabla^{\text{2}}\text{[Cdc20]}-r_{1}+r_{-1}-r_{3}+r_{-3}-r_{5}+r_{-5}$$

(8) $$\frac{\partial \text{[O-Mad2]}}{\partial t}{\text{=D}}_{\text{2}}\nabla^{\text{2}}\text{[O-Mad2]}-r_{1}+r_{-1}-r_{2}+r_{-2}-r_{4}+r_{-4}$$

(9) $$\frac{\partial \text{[Cdc20:C-Mad2] }}{\partial t}{\text{=D}}_{\text{3}}\nabla^{\text{2}}\text{[Cdc20:C-Mad2]}+r_{1}-r_{-1}+r_{3}-r_{-3}-r_{4}+r_{-4}+r_{5}^{2}-r_{-5}^{2}$$

(10) $$\frac{\partial \text{[Mad1:C-Mad2]}}{\partial t}={\text{D}}_{4}\nabla^{2}[\text{Mad1:C-Mad2}]-r_{2}+r_{-2}+r_{3}-r_{-3}$$

(11) $$\frac{\partial {\text{[Mad1:C-Mad2:Mad2}}^{\text{*}}\text{]}}{\partial t}{\text{= D}}_{\text{5}}\nabla^{\text{2}}{\text{[Mad1:C-Mad2:Mad2}}^{\text{*}}\text{] +}r_{2}-\;r_{-2}\;-r_{3}\;+\;r_{-3}$$

(12) $$\frac{\partial {\text{[Cdc20:C-Mad2:Mad2}}^{\text{*}}\text{]}}{\partial t}{\text{=D}}_{\text{6}}\nabla^{\text{2}}{\text{[Cdc20:C-Mad2:Mad2}}^{\text{*}}\text{]}+r_{4}-r_{-4}-r_{5}+r_{-5}.\;$$

where by *r_j_* denotes the reaction rate of reaction *j* and *r_−__j_* the rate of the inverse reaction. By adding a convectional term, the system can be extended to a *Reaction-Diffusion-Convection system*.

(13) $$\frac{\partial [C_{i}]}{\partial t}=\underset{\text{Diffusion}}{\underset{︸}{{\text{D}}_{i}\nabla^{2}[C_{i}]}}+\underset{\text{Convection}}{\underset{︸}{{\text{Q}}_{i}\nabla [C_{i}]}}+\underset{\text{Reaction}}{\underset{︸}{R_{j}(\{[C_{i}]\},\text{P})}}$$

The additional symbol Q*_i_* denotes the constant convection coefficient of species *i*. With the definition of the ∇ operator and its square, the full system looks like the following in Cartesian coordinates:

(13) $$\frac{\partial [C_{i}]}{\partial t}={\text{D}}_{i}\left(\frac{\partial^{2}[C_{i}]}{\partial x^{2}}+\frac{\partial^{2}[C_{i}]}{\partial y^{2}}+\frac{\partial^{2}[C_{i}]}{\partial z^{2}}\right)+{\text{Q}}_{i}\left(\frac{\partial [C_{i}]}{\partial x}+\frac{\partial [C_{i}]}{\partial y}+\frac{\partial [C_{i}]}{\partial z}\right)+R_{j}(\{[C_{i}]\},\text{P}).$$

#### 2.2.2. Model Assumptions

The mitotic cell is considered as 3-ball with radius *R*. The last unattached kinetochore is a 2-sphere with radius *r* in the center of the cell (see Table 1 for the species-dependent values of the radius).

We use a lattice based model, which implies that the reaction volume of the mitotic cell is segmented into equal compartments. The initial concentrations of Cdc20 and O-Mad2 are distributed randomly over all compartments of the mitotic cell. As Mad1:C-Mad2 and Mad1:C-Mad2:Mad2^*^ are only present at the kinetochore, their initial amount is located on the surface of the modeled 2-sphere. While reaction (1) can take place in any compartment, the reactions containing either Mad1:C-Mad2 or Mad1:C-Mad2:Mad2^*^ (reactions (2) and (3)) only occur at those compartments, attached to the kinetochore. In order to observe a more accurate spatial behavior of the “Mad2 template” model we do not consider any symmetrical restrictions. All boundary conditions are reflective in order that the amount of particles is conserved. We assume mass-action-kinetics for all reactions. As the diffusion constant of O-Mad2 is not known yet, we used parameter estimation to determine a suitable value (see Section 3). Active transport of O-Mad2 has been suggested to increase the inhibition of Cdc20:C-Mad2. Thus, we applied a convectional force to the species O-Mad2 which is direct proportional to the distance to the kinetochore with an unknown factor. We also used parameter estimation to determine the most realistic value for the convectional force term (see Section 3). All initial concentrations, reaction rates and environment parameter values are taken from literature (*cf.*Table 1). We did not model the Mad1:Mad2 complex formation and consider it as a preformed species in our model. We should point out here that this complex is a tetrameric 2:2 Mad1:Mad2 and not a monomer complex. However, considering single species from mathematical point of view would not make any difference in this case as long as we have one form in our model. All previous mathematical models have considered the same assumption to the template model (e.g., [28,32,33]).

#### 2.2.3. Numerical Simulation

We run our simulations using the Virtual Cell software [51]. We create the reaction volume according to the model geometry (*cf.* Section 2). Each dimension is divided into 51 parts, which results in 132.651 compartments in total. All parameters are set up consistent with the model assumptions. The system of PDEs with boundary and initial conditions is solved using the “Fully implicit finite volume with variable time-ste” method. This method employs Sundials stiff solver CVODE for time stepping (method of lines) [51]. The derivations, necessary for diffusion and convection, are computed numerically. We simulate the human system for 300 s which is sufficient to reach steady state (budding yeast took 5000 seconds), with a maximum time-step of 0.1 s and an absolute and relative tolerance of 1.0 *×* 10^−^^7^.

One simulation run takes between 2 and 20 h, dependent on the parameter-set. The time dependent concentration plots add up the amount of every species over all compartments and are generated with MatLab [52].

## 3. Results and Discussion

### 3.1. Quantitative Analysis of the SAC Model

The spindle checkpoint has to be fully active only abruptly after the cell enters mitosis and its activity must be maintained even with decreasing numbers of unattached kinetochores. After the last chromosome has established its connection to the mitotic spindle, the SAC must be rapidly deactivated. We showed in previous studies [28,29,30] that for fast checkpoint deactivation the destabilization of APC:inhibitor complexes might be required. Here, we focus on the formation of the Cdc20:C-Mad2 complex which is most certainly an APC:inhibitor.

Recently, it has been discussed in detail that the checkpoint activation is too slow with the measured *in-vitro* rate coefficients for the “Mad2 template” model in budding yeast. In agreement with this, non-spatial simulations of the model show an insufficient APC inhibition when using the measured yeast rate coefficients [32]. We could verify this hypothesis in our study. The inhibition of Cdc20 by O-Mad2 takes about 5000 min (data in supplement) which is way to long for an effect that has to occur nearly instantaneous. However, with a significant increase of all interaction rates the simulation of the model reaches steady state considerably faster. This supports that fast Cdc20:C-Mad2 formation indeed requires faster rates than the measured ones in budding yeast. With this result we will focus on the human model and use the budding yeast data only for comparison.

To establish checkpoint signaling within few minutes after entry into mitosis, the combined action of all kinetochores is required. However, when reducing the number of contributing kinetochores to one, *i.e.*, the situation right before the last attachment, checkpoint maintenance is no longer possible for the human and the yeast model (formation of Cdc20:C-Mad2 takes too long). When we looked closely to the species amounts at individual unattached kinetochores *in-silico*, we found the amount of O-Mad2 and activated O-Mad2^*^ molecules is very close to zero. When removing reaction (3) from the model (O-Mad2 binding to Mad1:C-Mad2), the formed amount of Cdc20:C-Mad2 was nearly zero. This indicates that the catalytic rate of Cdc20:C-Mad2 formation (reaction (1)) is not limiting. The majority of Cdc20:C-Mad2 is formed in vicinity of the kinetochore which is done by the catalyzing reaction (3). This implies that diffusive O-Mad2 alone cannot compensate the consumption of O-Mad2 at the kinetochores. Recent work [53] showed that the human protein Tpr binds Mad2 in the region of the mitotic spindle even in the absence of microtubules. Mad2-localization to the spindle region was formerly attributed only to dynein binding. Hence, we speculate that O-Mad2 might also be actively transported towards the spindle mid-zone to increase restoration of O-Mad2 at unattached kinetochores.

Note that the combined contribution of all kinetochores may be sufficient to activate the checkpoint even without such a mechanism, especially in combination with other mechanisms like Cdc20 sequestration by Emi1 (only meiotic, we do not need it here) [54,55] or preformed MCC [34]. Taken together, with our current given knowledge, SAC control alone is insufficient to guard chromosome attachment.

### 3.2. Reaction-Diffusion System of the “Mad2 Template” Model

**Figure 1:**
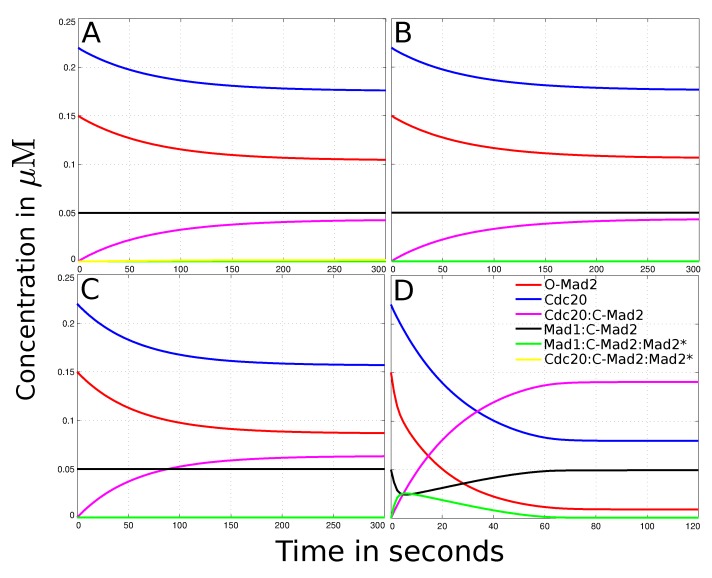
Dynamical behavior of the spatial human SAC model. The figures show the total concentrations over time for every species with different parameter sets. (**A**) Outcome of the simulated “Mad2 template” model (experimental interactions, diffusion rates and no convection, *cf.*Table 1). It takes about 5 min to reach steady state. O-Mad2 and Cdc20 are reduced to 70% and 80% of their initial amount, respectively. Cdc20:C-Mad2 has a concentration of 4.3 *×* 10^−^^2^*µM* (20% of Cdc20) in the steady state. According to literature, this amount is not enough and its formation too slow to dissipate the “wait anaphase” signal. Mad1:C-Mad2 and Mad1:C-Mad2:Mad2^*^ do not change their concentration over time and thus do not contribute to the formation of Cdc20:C-Mad2 significantly; (**B**) The results of the reduced “template model” (slow diffusion constant and deleted reactions (4) and (5)) do not deviate significantly from the full model, *cf.*panel A. Neither the amount of Cdc20:C-Mad2 is increased nor the time it takes to reach steady state decreased. Thus, the autocatalytic loop, especially the formation of the Cdc20:C-Mad2:Mad2^*^ complex, has no influence on the model and can be omitted; (**C**) The simulation of the model with a 4-fold higher diffusion constant of O-Mad2 (D*_i_*= 20 *µ*m^2^s^−^^1^). The species’ dynamical behavior is qualitative the same as in the standard simulation (panel B). However, with a higher diffusion constant the final amount of Cdc20:C-Mad2 increased to 6.3 *×* 10^−^^2^ *µ*M (29% of Cdc20), whereby O-Mad2 and Cdc20 are decreased to 57% and 71% of their initial concentration, respectively; (**D**) The simulation of the model with an active transport of O-Mad2 (*cf.* Equation (13)). It takes 1*−*2 min to reach steady state. The active transport of O-Mad2 towards the kinetochore promotes the rates of reactions (2) and (3). Thus, the inhibition level of Cdc20 raises to 60% of its total amount. Mad1:C-Mad2 and Mad1:C-Mad2:Mad2^*^ catalyze the formation of Cdc20:C-Mad2 and reduce free Mad2 to 11% of its initial concentration.

**Figure 2:**
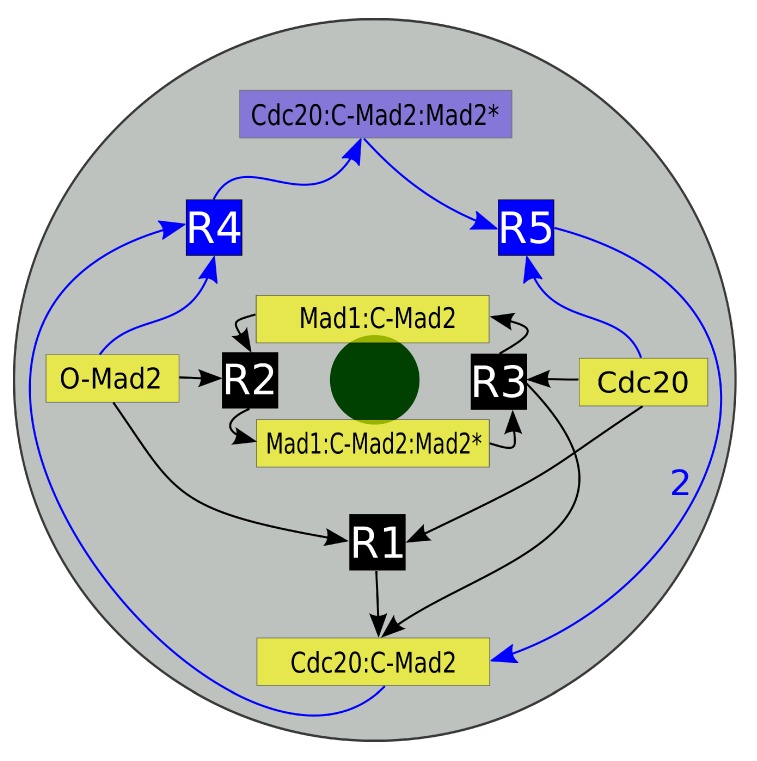
Schematic diagram of the “Mad2 template” model. Depicted is a projection of our mitotic cell. The gray disk corresponds to the cell while the green disk presents the last unattached kinetochore. Inscribed is the reaction network of the “Mad2 template” model with species localization. The core model consists of five species (yellow boxes) and their three reactions (black boxes R1, R2 and R3). The full model furthermore contains the amplification pathway, which is symbolized by the blue boxes (Cdc20:C-Mad2:Mad2*, R4 and R5). The arrows’ directions indicate whether a species is a reactant or a product. Mad1:C-Mad2 and Mad1:C-Mad2:Mad2^*^ are localized predominantly in the vicinity of the kinetochore and the later catalyzes reactions R2 and R3. The autocatalytic amplification reactions of Cdc20:C-Mad2 occur in the cytosol (see Section 2 for details).

Ibrahim *et al*. [28] found that in the “Mad2 template” model the amplification by the autocatalytic loop (reactions (4) and (5)) is vanishing if the reaction rate constants are low. To make sure that these results are still valid with our spatial reaction-diffusion system, we execute a simulation by integrating the autocatalytic amplification of Mad2. As the diffusion rate of Mad2 is unknown, we vary it between 0–50 *µ*m^2^s*^−^*^1^. However, the changes when including the reactions of the autocatalytic loop are not perceptible when compared to the basic “template model” (*cf.* Figure 1A,B). This is because direct binding (reaction (1)) dominates Cdc20 sequestering [33], while kinetochore dependent catalysis (reactions (2) and (3)) becomes increasingly important during the inhibition mechanism. Thus, we remove the reactions (4) and (5) from the network (*cf.* Figure 2 black graph) as they have no effect on the outcome of the simulation in steady-state.

**Figure 3:**
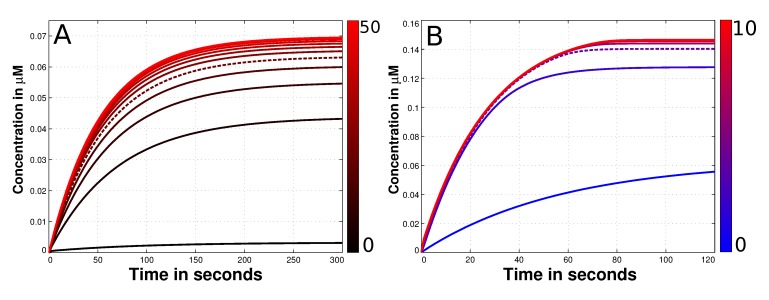
Diffusion and convection parameter estimation of the species O-Mad2. (**A**) Depicted is the time dependent concentration of Cdc20:C-Mad2 with different diffusion constants of O-Mad2. The variation of this rate is shown next to the plot with the color gradient. The black curve denotes a slow diffusion (0 *µ*m^2^s^−1^) while red curves present fast diffusion values (up to 50 *µ*m^2^s^−1^). At the end of the simulations it is a maximum of 0.07 *µ*M of the Cdc20:C-Mad2 complex formed. If the diffusion constant Di is greater than 20 *µ*m^2^s^−1^ no significant alteration can be observed in terms of produced Cdc20:C-Mad2. Thus, we used this value for all other simulations (dashed line); (**B**) presented is again the time dependent concentration of Cdc20:C-Mad2. This time the convection rate of O-Mad2 is varied according to the color gradient. The blue curve has no convection and consequently is the same like the dashed plot in panel A. Red curves have a convection up to 10 *µ*ms^−1^. Exceeds the convection 4 *µ*m^2^s^−1^, no alteration of the formed Cdc20:C-Mad2 can be observed in steady state. As a result we used this value for the force, transporting O-Mad2 towards the spindle mid-zone.

Using only the reduced SAC model we figure out the most suitable value for the diffusion constant of free Mad2. Therefore, we vary the unknown diffusion constant of O-Mad2 for wide range between 0–50 *µ*m^2^s^−^^1^. The amount of formed Cdc20:C-Mad2 and time until the simulation reaches steady state are our measurements for the quality of the SAC model. We found no major effects if the diffusion exceeds 20 *µ*m^2^s^−^^1^(see Figure 3A). Since the highest diffusion rate in the model is 19.5 *µ*m^2^s^−^^1^ (from Cdc20), we mainly focus our comparison on high diffusion rate of Mad2 (20 *µ*m^2^s^−^^1^) versus slow diffusion rate (5 *µ*m^2^s^−^^1^). The result of this comparison shows only minor effects for the formation of the Cdc20:Mad2 complex (see Figure 1B,C). This outcome is consistent for both human and budding yeast models (yeast data in supplement, due to its effect is even less).

### 3.3. Mad2 Active Transport towards Spindle Mid-Zone

We propose an active transport of O-Mad2 towards the spindle mid-zone using convection (see Section 2). This results in a so called reaction-diffusion-convection system. As the magnitude of the convection is completely unknown (symbol Q*i *in Formula (13)), we estimate the best value regarding the amount of formed Cdc20:C-Mad2. We run simulations with a convection rate constant between 0–10 *µ*ms^−1^. Our results show that any convection constant larger than 4 *µ*ms^−1^ has no significant influence to the formation of the Mad2:Cdc20 complex (*cf.*. Figure 3B). Thus, we use a convection rate of (${\vec{Q}}_{i}=-4.0\times {\vec{e}}_{i}\;µ{\text{Ms}}^{-1}$) for O-Mad2 to achieve an active transport towards the midpoint of the cell.

With the human model settings (*cf.* Table 1), this transport of O-Mad2 towards the spindle mid-zone greatly enhances the formation of Cdc20:C-Mad2 (see Figure 1D). Interestingly, the level of Cdc20 inhibition is even higher with the active transport model (about three times the amount of the standard model). Moreover, the time required to reach steady state in the active transport model was very fast and near to reality (about 1–2 min, *cf.* Figure 1D). In comparison it takes more than twice as long in the model without an active transport (about 5 min, *cf.* Figure 1A). As expected, with the budding yeast model settings (see Table 1) no significant improvements are recorded (data shown in supplement). This is due to the fact that budding yeast cells are relatively small in size and diffusion alone can provide enough O-Mad2 at the kinetochore.

Taking together, our *in-silico* data suggest that an active transport of O-Mad2 towards the spindle mid-zone may increase the efficiency of the “Mad2 template” model in human cells. Activated Mad2 inhibits significantly more Cdc20 via the Cdc20:C-Mad2 complex then in other models, without any convectional forces.

## 4. Conclusions

In eukaryotic cells, the mitotic spindle assembly checkpoint is an important regulatory mechanism for accurate chromosome segregation. The checkpoint guarantees that each chromosome has established its attachment to the spindle apparatus before sister-chromatid separation is initiated in anaphase. Consequently, its failure has been implicated in tumorigenesis [10,56]. Quantitative analysis and computational modeling are very important tools to elucidate how such elaborate systems work. So far, mathematical models have helped to enlighten kinetochores structure and with that the mitotic checkpoint mechanism [26,27,28,29,30,32,33,57,58,59,60,61,62,30,32,33,57]. These models helped to pinpoint advantages and problems of putative regulatory mechanisms. However, the conclusion of these models is that SAC mechanism alone is insufficient to guard chromosome attachment.

In this paper, we have analyzed a spatial SAC model from two different organisms, human and budding yeast Saccharomyces cerevisiae, with different parameter sets. We first verified the hypothesis that the autocatalytic loop is not contributing to the formation of Cdc20:C-Mad2. Based on this result, we reduce the model and determine the diffusion constant of free Mad2 as 20 *µ*m^2^s^−^^1^. Even with a higher diffusion the required Mad2 at the kinetochore could not be covered. Therefore, we propose an additional mechanism for SAC functioning which is an active transport of O-Mad2 towards the spindle mid-zone in human cells. The simulation of our model indicates that this mechanism greatly enhances Cdc20:Mad2 formation. The experimental validity of this prediction is pending and needs to be investigated in the future. The outcome of the yeast simulations are consistent with the *in-vitro* experiments, if we assume 100-fold higher interaction rates.

This reveals that the model for the budding yeast is incomplete or in a lack of experimental findings. In contrast to the human setup, the needed Mad2 at the kinetochore is covered alone by diffusion, as budding yeast cells are way smaller than human cells. Future model validation in an integrative approach, theoretical and experimental, will help to reveal the SAC mechanism on the molecular level. An example for an analogous mechanism is the spindle position checkpoint [63,64]. We anticipate that a systems biological approach of the SAC mechanism will serve as a basis to integrate future findings and evaluate novel hypothesis related to checkpoint architectures and regulation.

## Supplementary Materials

Supplemenatry figure can be found at http://www.mdpi.com/1422-0067/15/10/19074/s1.

## Supplementary Files

Supplementary File 1
