# Supplementary material for: Active Transport Can Greatly Enhance Cdc20:Mad2 Formation
Source: Int J Mol Sci. 2014 Oct 21;15(10):19074–91. doi: 10.3390/ijms151019074 (PMC4227261; doi:10.3390/ijms151019074)

Supplementary Information

Figure S1. Dynamical behavior of the spatial budding yeast SAC model. The figures show the total concentration over time for every species with different parameter sets. (**A**) Simulation results of the standard model for a budding yeast cell (experimental interaction, discussion rates and no convection, cf. Table 1). The steady state is reached in 5000 min, which is exceptionally long. O-Mad2 and Cdc20 concentrations are reduced to 70% and 40% of the initial amount, respectively. Cdc20:C-Mad2 has a concentration of 6.0×10^{-2} µM (60% of Cdc20) in the steady state while Mad1:C-Mad2 and Mad1:C-Mad2:Mad2* do not change their concentration over time significantly which is very close to zero; (**B**) Simulation with a 100-fold increase of all reaction rates. Now, the steady state is reached after 30 min which can be count for acceptable time. No qualitative change of the Cdc20:Mad2 level was observed; (**C**) Simulation with solely increasing the formation of Cdc20:C-Mad2 interaction rate (3) 100-fold higher. Steady state is reached about 10-times faster (500 min) than in the standard simulation (**panel A**). The concentration of Cdc20 nearly goes to 0 µM while O-Mad2 is reduced to ≈50% of its initial amount. Cdc20:C-Mad2 goes up to a final concentration of 0:1_M; (**D**) The same setup as shown in **panel C**, with a 104-fold increased reaction rate (3). Again the steady state is reached faster (about 12 min), while the formation level of Cdc20:C-Mad2 is almost the same.

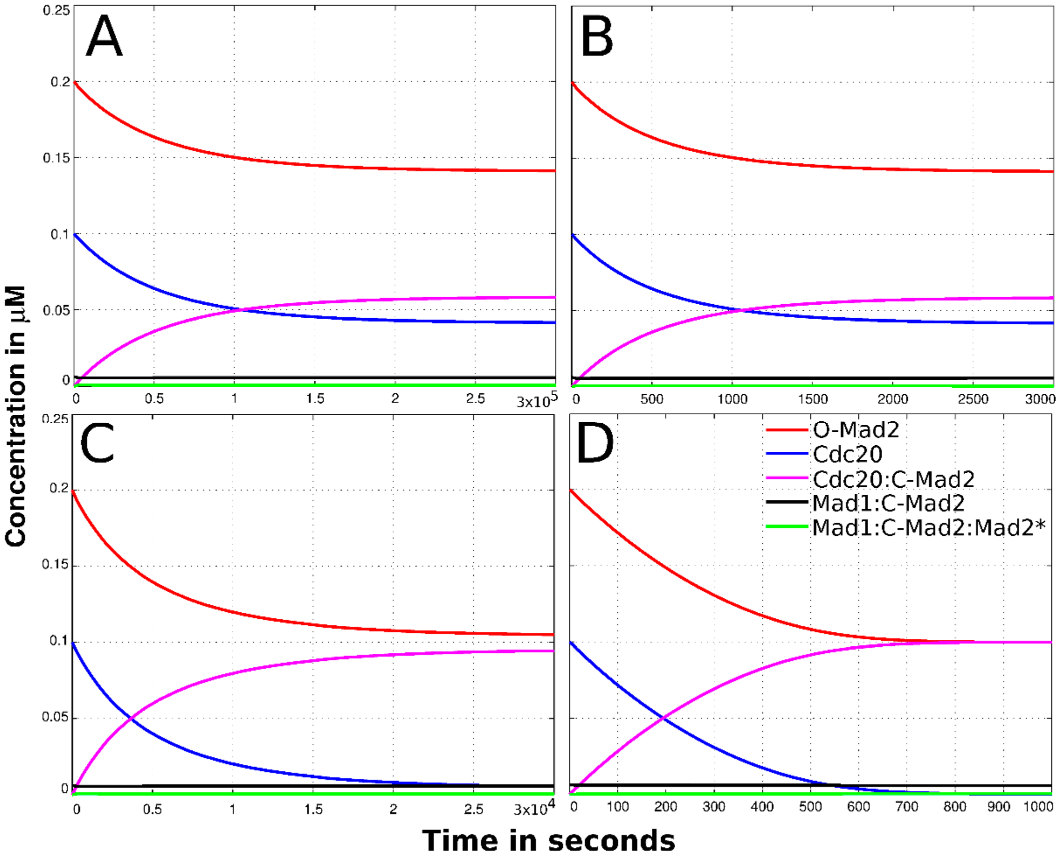

Supplement: Supplementary File 1 [file ijms-15-19074-s001.pdf]
